# Towards a remote sensing-based assessment of carbon emissions from peatlands

**DOI:** 10.1038/s41598-025-15293-1

**Published:** 2025-10-01

**Authors:** Pouya Ghezelayagh, Andrzej Kamocki, Piotr Banaszuk, Mateusz Grygoruk

**Affiliations:** 1 Centre for Climate Research, Warsaw University of Life Sciences-SGGW, Nowoursynowska 166, Warsaw, 02-787 Poland; 2 Faculty of Civil Engineering and Environmental Sciences, Białystok University of Technology, ul. Wiejska 45A, Białystok, 15-351 Poland

**Keywords:** Peat degradation, Subsidence, Soil organic carbon (SOC), Bulk density, Oxidation, Climate change, Environmental sciences, Hydrology, Natural hazards, Climate change

## Abstract

Among greenhouse gases-generating sources, biosphere sources from natural carbon (C) reservoirs play a significant role. A vital component of the biosphere is peatlands—the largest natural terrestrial carbon storage on the earth. Peatlands function as both C sink and C source, showing their pivot role in mitigating GHGs. Releasing C results from peat oxidation—the decomposition of organic matter in the peat. This decomposition reduces the volume of peat and, hence, causes subsidence. This study introduces an exclusive remote-sensing-based framework for estimating carbon emissions from peatlands using subsidence rates. This framework integrates peat properties—bulk density and soil organic carbon—with the oxidated peat subsidence, which refers to the proportion of subsidence attributed to the oxidation process rather than shrinkage. Achieving a fully remote-sensing-based approach promises time-effective, cost-effective, and consistent C emission monitoring even in unreachable places in peatlands, addressing the critical need for global climate change mitigation strategies. However, this achievement requires collaborative efforts among researchers to implement it in other sites to improve dataset accuracy for each parameter. By improving this framework, the scientific community can pave the way for robust, large-scale assessments of peatland C emission.

**Supplementary Information:**

The online version contains supplementary material available at 10.1038/s41598-025-15293-1.

## Introduction

Climate change has risen as one of the highest global concerns in contemporary times^1,2^. The increasing levels of greenhouse gases (GHGs) in the atmosphere have resulted in ascending temperatures and many damaging environmental consequences^3–5^. GHGs are atmospheric components that inhibit the escape of the Sun’s reflected electromagnetic radiation from Earth’s surface back into space^6,7^. These gases—primarily carbon dioxide (CO₂), methane (CH₄), nitrous oxide (N₂O), and water vapor (H₂O)—function analogously to the glass walls of a greenhouse, trapping heat within the atmosphere^8,9^. Among GHGs, the ones containing carbon (C) play a significant role as the dominant element in this heat-retention process^10,11^. While GHGs-generating sources—such as fossil fuel combustion, cement production, and other industrial processes—are the largest, biosphere sources also play a significant role^12^. Land biogenic GHG fluxes arise from natural carbon reservoirs and are shaped by both natural variability and human-induced disturbances^13,14^. A vital component of the biosphere is peatlands—terrestrial wetland ecosystems where waterlogged conditions inhibit the complete decomposition of plant material, resulting in the gradual accumulation of peat over time^15,16^. Peatlands cover less than 3% of the Earth’s surface but store more C than the combined biomass of the world’s tropical rainforests and over half of all the C currently in the atmosphere^17–21^. To affirm the great importance of peatlands, it suffices to say that northern boreal and temperate peatlands are the largest natural terrestrial carbon storage on the earth^22,23^. Although the ability of peatlands to function as a C sink rather than a source shows their pivot role in mitigating GHGs^24–27^ the degradation of peatlands transforms this role into a dramatically harmful one, resulting in a substantial increase in C emissions^28,29^. Peatland degradation leads to a reduction in peat volume, which manifests as subsidence—the downward movement of the ground surface. Therefore, given the direct relationship between degradation and C loss, as well as the link between degradation and subsidence, the subsidence rate can serve as a proxy for estimating C emission from peatlands^30,31^.

Using subsidence as a proxy requires a clear understanding of its driving factors in peatlands^32,33^. Subsidence indicators analyzed in a seasonal pattern can be used in quantifying bog breathing process. Subsidence is primarily driven by oxidation and shrinkage^34–36^. Oxidation involves the decomposition of organic matter in the peat, releasing C into the atmosphere^37,38^. Shrinkage, on the other hand, is associated with the loss of water content within the peat, which translates to compaction^39,40^.

Several studies have utilized peat subsidence as a proxy for estimating C emissions. This estimation requires three key components: the oxidation component (α), peat properties (Cv), and the subsidence rate (Δh_s_). Since the amount of C emitted from degraded peatlands is caused by oxidation processes, distinguishing it from the shrinkage component is essential, and this is where α comes into play. Sari, et al.^41^ Khodaei, et al.^42^ Hayati, et al.^43^ Erkens, et al.^44^ Zhou, et al.^45^ Hooijer, et al.^46^ Mos, et al.^47^ Anshari, et al.^48^ Couwenberg, et al.^49^ and Wösten, et al.^50^ have estimated CO_2_ emissions based on the assumption that oxidation contributes a substantial portion of subsidence, attributing approximately 60–100% (α = 0.6–1) of the total subsidence^51,52^. On the other hand, Grønlund, et al.^53^ reported that in Norwegian peatlands, oxidation accounted for 30–50% of total subsidence, which aligns with findings from Dutch peatlands, where oxidation contributions ranged from 35 to 50%, as noted by Schothorst^54^. These values show the lack of transparency in determining oxidation components, confirming the urgent need to adopt a unified approach. Therefore, one of the primary goals of this study is to propose this unified approach inspired by a global meta-analysis research conducted by Ma, et al.^55^.

The bulk density (BD) and soil organic carbon (SOC) content—the property of peat (Cv)—are the other important parameters in estimating C loss from subsidence rates. These parameters are typically obtained through laboratory analysis of soil samples; however, the challenges of obtaining accurate field measurements have often led to their being overlooked. As a result, many studies commonly use constant values of 80 kg/m³ for BD and 55% for SOC. Agus, et al.^49^ express that the BD and SOC range 20–300 kg/m^3^ and 18–58%, which implies ranging from 3.6 to 174 kg-C/m^3^ for Cv. So, this study also aims to improve these parameters by utilizing the dataset provided by Loisel, et al.^56^ for northern boreal and temperate peatlands, and the one introduced by Agus, et al.^51^ for tropical peatlands.

Recent advancements have made it possible to estimate peat subsidence rates (Δh_s_) using remote sensing (RS), particularly the Interferometric Synthetic Aperture Radar (InSAR) technique^57–59^. Ghezelayagh, et al.^57^ demonstrated the use of InSAR for peatland ecosystems, employing Sentinel-1 satellite processed via Alaska Satellite Facility (ASF) OnDemand InSAR cloud computing products. This approach includes systematic error reduction to improve interferogram quality, accounting for spatial and temporal baseline, atmospheric condition, and ground cover.

After proposing a remote-sensing-based framework, this study evaluates C emissions by applying it to the Biebrza Valley in Poland and validating each parameter against field survey-based data. We believe this study represents a significant step toward developing a more accurate, fully remote-sensing-based framework for estimating carbon emissions from peatlands. With high accuracy, such a framework would facilitate timely and consistent C emission monitoring and provide cost-effective, spatially distributed, and easily accessible data. This enables us to do large-scale assessments of peatland carbon dynamics and emissions. Achieving this goal requires a collective effort and effective communication among experts in this domain who have access to more in-situ data.

## Results and discussions

### Framework description

**Fig. 1:**
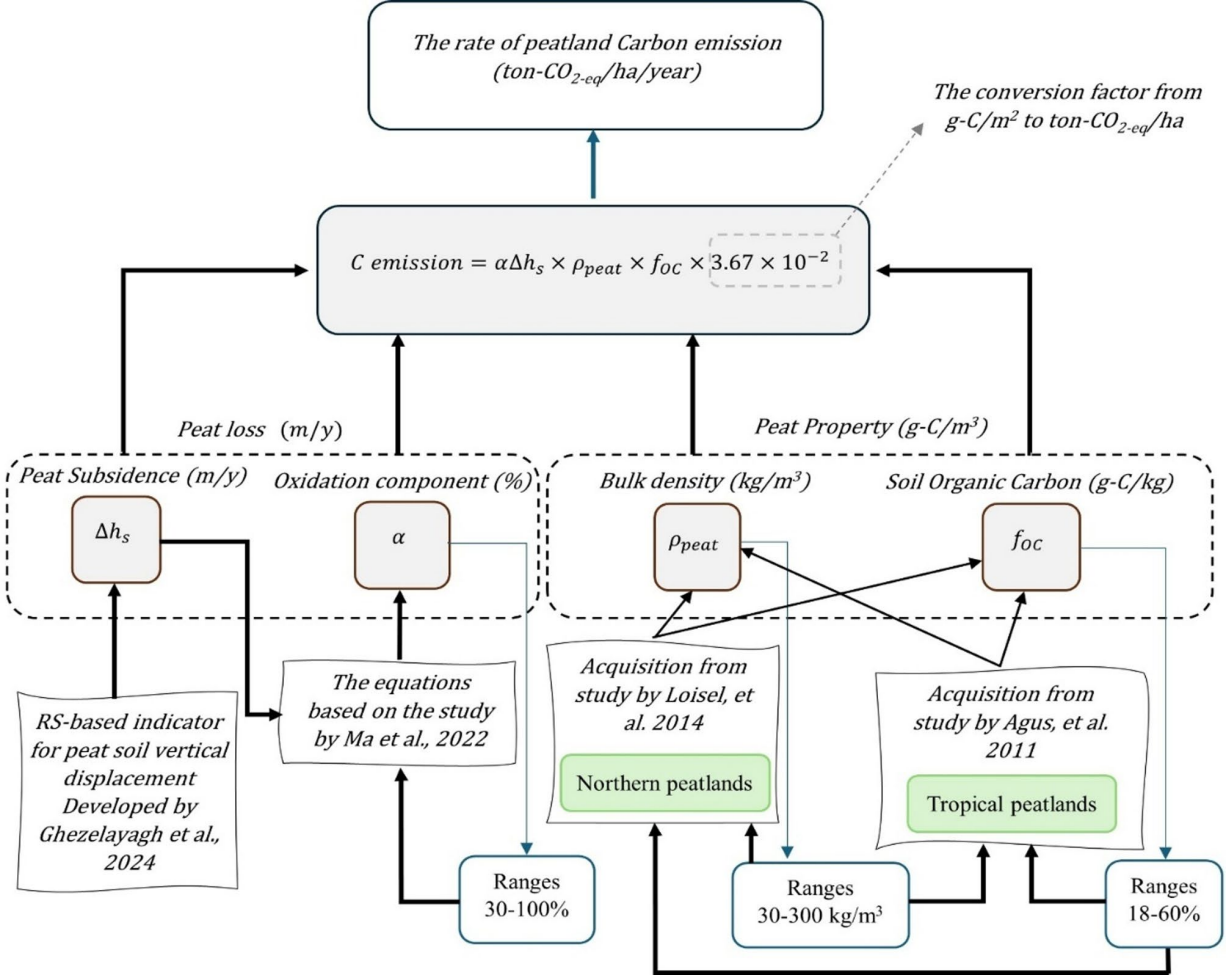
Remote sensing (RS)-based framework for estimating C emission (CO_2−eq_/ha/year). The estimation is based on the peat loss (αΔh_s_) and peat properties (C_v_), where Δh_s_ represents the subsidence rate, α the oxidation component, $\:{\rho\:}_{peat}$ bulk density and $\:{f}_{OC}$ soil organic carbon content.

Figure 1 presents the framework for estimating C emissions based on the amount of C lost by employing peat property (C_v_) and the loss of peat $\:{(\alpha\:\varDelta\:h}_{s})$—the proportion of subsidence is caused by the oxidation process rather than shrinkage. This approach requires four key parameters: subsidence rate (m/y), oxidation component (%), BD (kg/m^3^), and SOC content (g-C/kg) (Fig. 1). In the following section, we detail the methods for acquiring these parameters without relying on field survey data, enabling us to achieve a fully RS-based approach to C emission estimation. The results for each parameter were validated against field survey-based data to assess their validity.

**Table 1:** The equations for determining oxidation components in Northern peatlands using rate of subsidence (cm/year).

Peatland type		Equations
Northern boreal Peatlands	***Agriculture***	α$\:\:=2.15+12.05\text{ln}(\sqrt[-0.58]{\frac{{\varDelta\:h}_{s}}{13.95}})$
	***Forestry***	α$\:\:=2.15+12.05\text{ln}(\sqrt[-0.83]{\frac{{\varDelta\:h}_{s}}{5.36}})$
	***Grassland***	*α* $\:\:=2.15+12.05\text{ln}(\sqrt[-0.36]{\frac{{\varDelta\:h}_{s}}{5.55}})$
*Tropical Peatlands*	***Agriculture***	*α* $\:\:=2.15+14.36\text{ln}(\sqrt[-0.37]{\frac{{\varDelta\:h}_{s}}{6.63}})$
	***Forestry***	

### The rate of subsidence (Δh_s_)

**Fig. 2:**
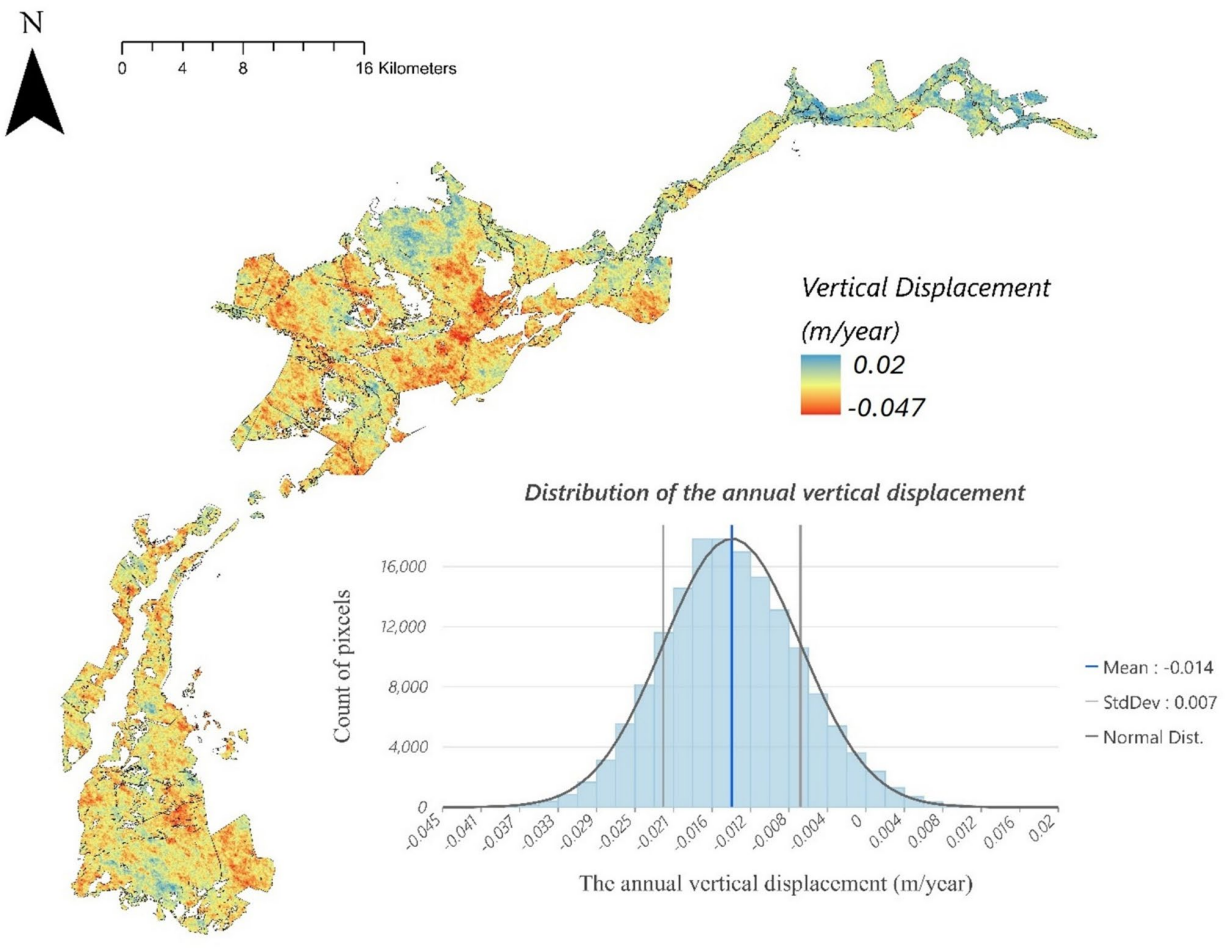
Spatial distribution of annual vertical displacement rates in the Biebrza Valley. Negative values indicate peat subsidence, while positive values represent peat accumulation. These rates were derived using the remote-sensing-based indicator proposed by Ghezelayagh, et al.^57^.

Figure 2 shows the spatial distribution of vertical displacement rates across the study area obtained by implementing the remote-sensing-based indicator proposed by Ghezelayagh, et al.^57^. The results reveal that the range of annual vertical displacement in the case study is from − 4.5 (subsidence) to + 2 cm/year (accumulation). The distribution histogram chart indicates that this case study’s mean annual subsidence rate is 1.4 cm (an average of 0.014 ± 0.007 m per year). However, for C emission estimates, only areas experiencing subsidence (negative vertical displacement) are considered, as emissions are directly linked to the oxidation of peat during subsidence. Therefore, this approach quantifies the subsidence rate rather than the total vertical displacement, as only subsidence contributes to carbon emissions. More detail is provided in supplementary material 1 and Table S1.

### Oxidation component (α)

α represents the fraction of subsidence attributed to the oxidation of organic matter in peatlands, rather than processes like shrinkage or consolidation. Estimating α is crucial for determining the C emissions associated with peat subsidence. In this study, α was acquired by the following equations derived from a global meta-analysis conducted by Ma, et al.^55^. Table 1 is derived by manipulating equations from Ma et al.‘s study (see Methods).

**Fig. 3:**
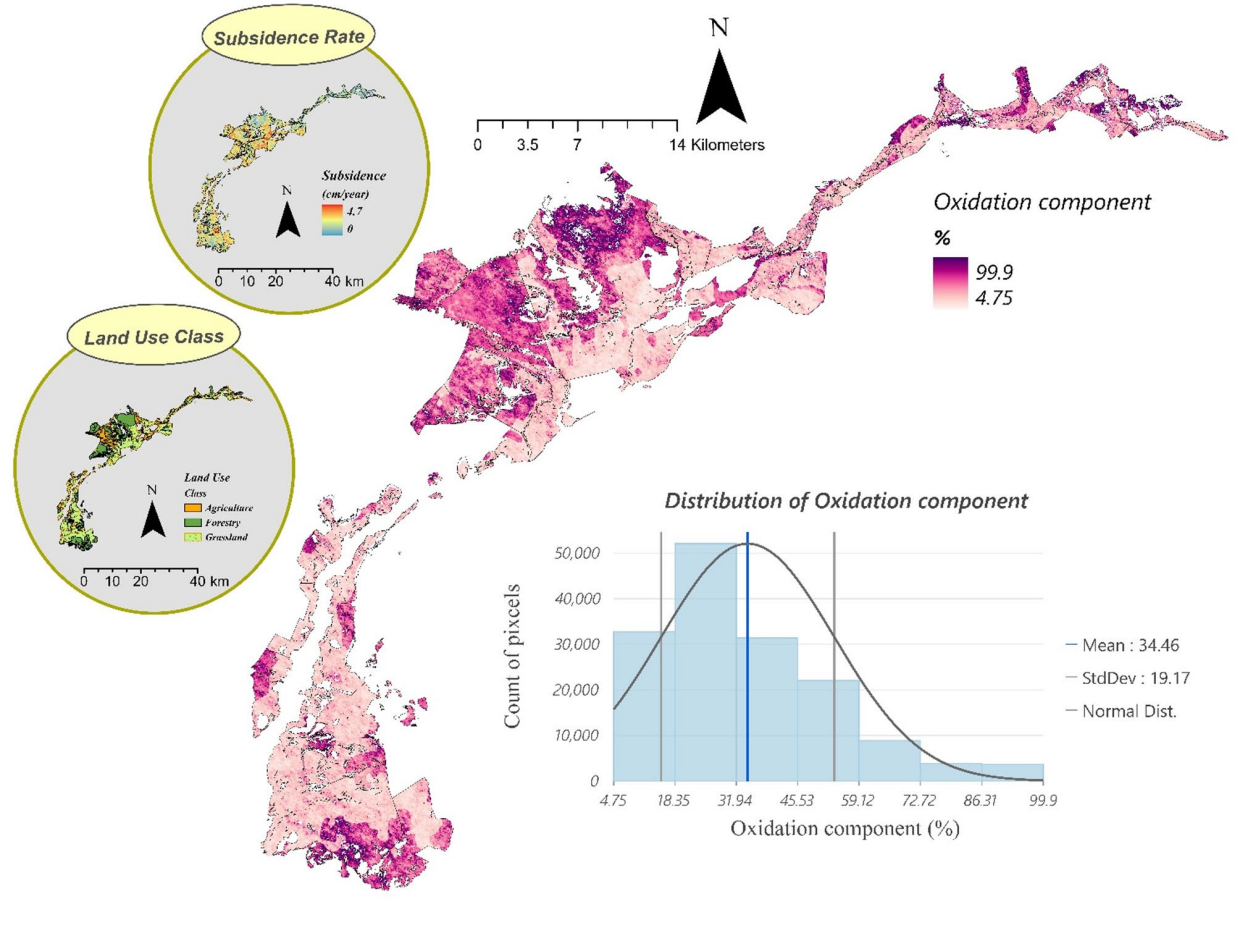
Spatial distribution of the oxidation component (%) across the Biebrza Valley, showing the proportion of peat subsidence attributed to oxidation processes. Land use classes include agricultural land, which consists of permanent meadows and pastures; grassland, which refers to extensively used meadows; and forest.

The results (Fig. 3) indicate that the oxidation component varies spatially across the study area due to differences in land use. On average, α was 34.46% (± 19), meaning approximately 35% of the subsidence is attributable to oxidation. This aligns with findings from previous studies in Dutch and Norwegian peatlands, which reported α values ranging from 0.3 to 0.5 (Schothorst^55^ and Grønlund, et al.^53^).

The spatial distribution of α shows higher values in areas with agricultural land use (including permanent meadows and pastures), indicating that peat oxidation is more pronounced under such conditions. Conversely, lower α values are observed in grassland (including extensively used meadows), areas with higher moisture retention or minimal anthropogenic interference.

### Peat property (BD and SOC)

**Fig. 4:**
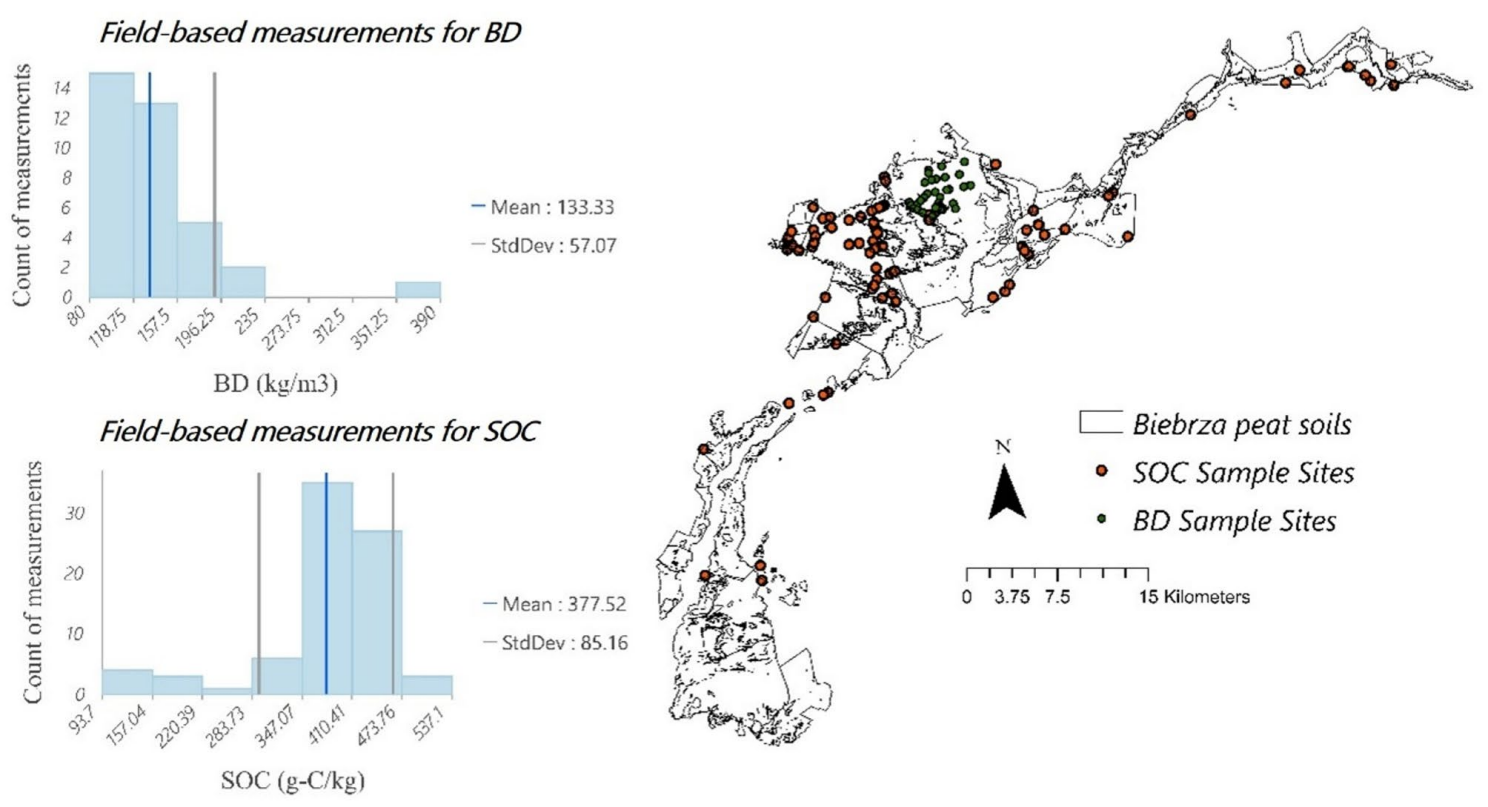
Field-measured data for BD and SOC content in the Biebrza Valley. The data were utilized to validate the BD and SOC estimates derived from the RS framework approach.

BD and SOC content directly determine the amount of carbon stored in peat soils and the carbon released during oxidation. In this approach for northern boreal and temperate peatlands, BD and SOC are acquired based on an in-suit dataset introduced by Loisel, et al.^56^. The dataset provides a table in which the location of the interested areas can acquire these parameters (see methods). According to Table 3 and taking into account the location of Biebrza in continental Europe, the BD value in the table is 0.120 ± 0.139 g/m^3^, ~ 120 kg/m³ (obtained from 410 field measurements), while SOC content is 38.9 ± 1.3% (from 60 sites), which means 389 g-C/kg. These results show that the value of 46.7 kg/m^3^ for C_v_ aligns with values typically reported for temperate peatlands, ranging from 46 to 82 kg/m³^51^. To ensure accuracy, the estimated BD and SOC values were validated against field survey data collected from the case study (Fig. 4).

To assess the deviation in SOC estimates, we first calculated the difference between their central values (389 ± 13 and 377.5 ± 85.2 g-C/kg), yielding a difference of 11.5. Next, we determined the combined standard deviation by taking the square root of the sum of the squares of the individual standard deviations (13 and 85.2). By normalizing the difference between the central values using the combined standard deviation, we obtained a normalized difference of approximately 0.133. By adopting a similar approach for BD (133.3 ± 57 and 120 ± 139 kg/m³), this value was calculated at 0.0865. Additionally, to be more confident in assessing BD deviation, we also used in-situ data reported by Gnatowski, et al.^60^ from the Biebrza River Valley, based on 87 samples, which indicated an average bulk density of 132 ± 38 kg/m³. The normalized difference for BD was approximately 0.125.

The slight difference between the central values relative to their combined uncertainties, 0.133 for SOC and 0.0865 for BD, indicates that the estimated and measured values are in good agreement.

### Estimation of C emission

**Fig. 5:**
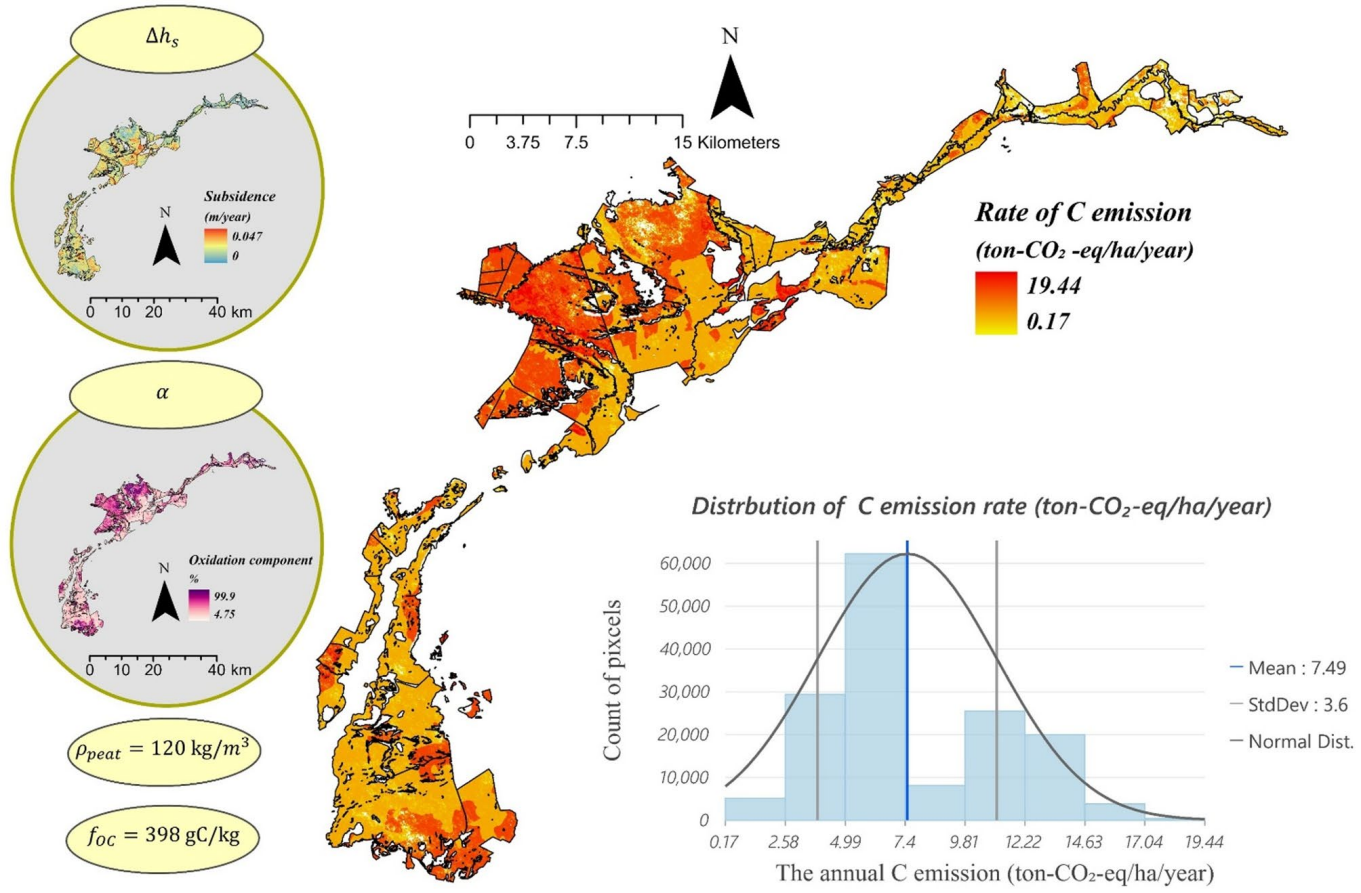
The distribution of the C emission rate in the Biebrza Valley (ton-CO_2-eq_/ha/year) estimated through rate of subsidence (m/year), oxidation component (%), Bulk density (kg/m^3^), and Soil organic carbon content (g-C/kg).

The C emission rate is expressed in tons of CO_2-eq_ per hectare per year. This metric provides a unified approach by converting all carbon emissions to CO₂, allowing for better comparison with other studies, as most research in this field reports emissions in terms of CO₂. Figure 5 illustrates the C emission rate and its distribution across the case study. The histogram shows that the C emission rate in Biebrza Valley is 7.49 ± 3.6 ton-CO_2-eq_/ha/year.

Moreover, the common approach for estimating CO_2-eq_ emissions from peatlands using subsidence as a proxy assumes constant values for BD (80 kg/m³) and SOC content (550 g-C/kg), with an oxidation component range of 30–100%. Using this approach, we estimated CO_2-eq_ emission rates of 14.49 tons/ha/year, assuming a 60% oxidation rate commonly used in previous studies (see introduction). Several studies have employed this approach: Khodaei, et al.^42^ estimated C emissions in Sweden at approximately 6.7 ton-CO_2−eq_/ha/year based on a 0.9 cm annual subsidence rate; Leifeld, et al.^61^ reported a range of 9.2–20.2 ton-CO_2−eq_/ha/year in exchange for subsidence rates of 0.8–1.6 cm/year in European’s temperate fens.

**Fig. 6:**
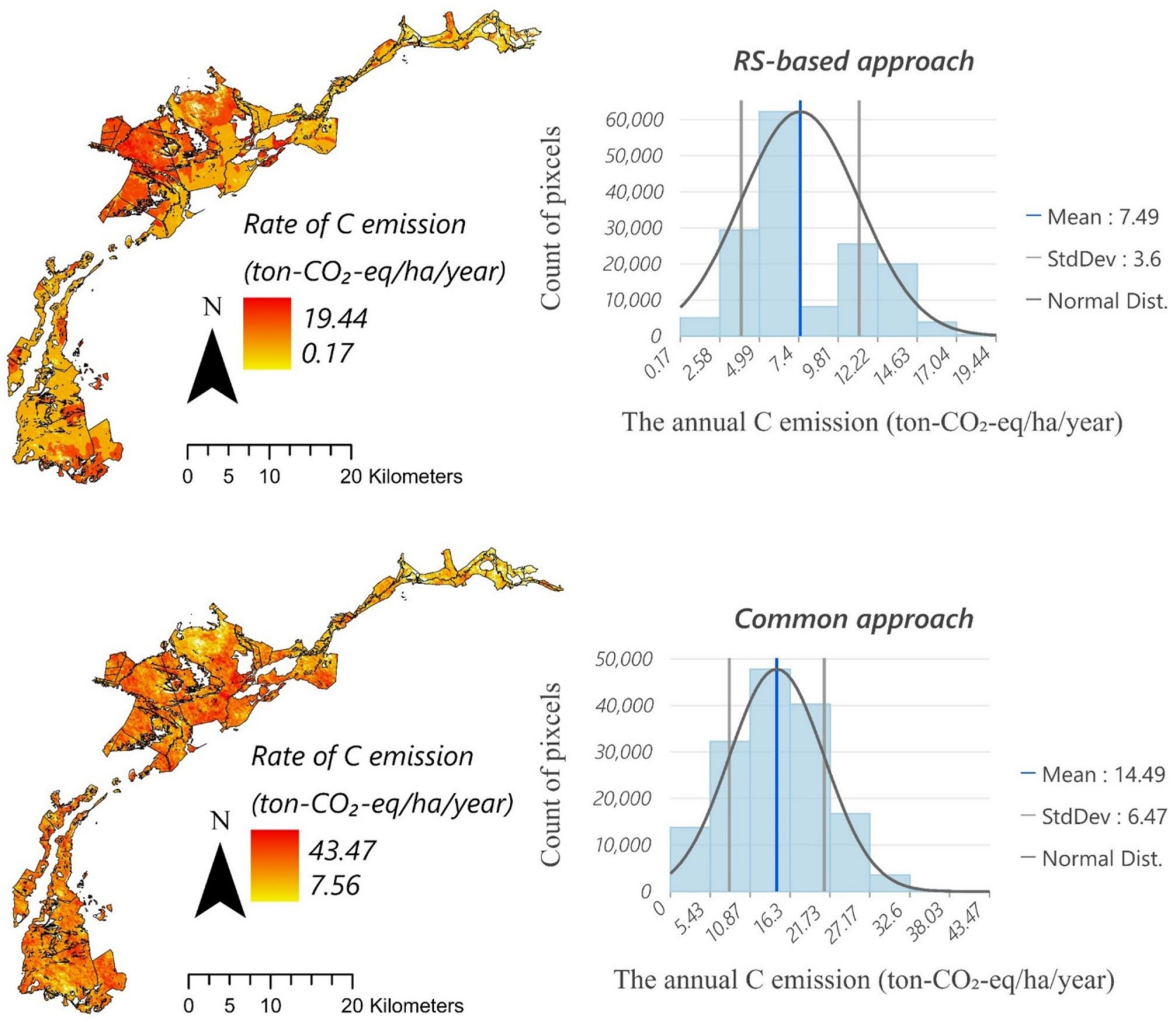
The comparison of C emission estimation between the RS-based and the common approach.

However, the estimation derived from the proposed framework is more consistent than the common approach with the findings of Wilson, et al.^62^ who reported C emissions from peatlands in Canada, the Republic of Ireland (ROI), the United Kingdom (UK), and Fennoscandia to be approximately 6.2 (± 0.47) ton-CO_2−eq_/ha/year for industrial extraction sites and 6 (± 0.44) ton-CO_2−eq_/ha/year for domestic extraction sites in the ROI and UK. These values are notably lower than the IPCC^63^ Tier 1 default emission factor of 10.3 (± 1.7) ton-CO_2−eq_/ha/year. Wilson et al.’s study highlights the necessity of refining regional emission factors to improve the accuracy of greenhouse gas inventories​. Höper, et al.^64^ found that rates of carbon emissions from peatlands in Central European countries, including Poland, Germany, the Netherlands, and Sweden, range from 15 to 17 ton-CO_2−eq_/ha/year. Boreal fens under grass and barley cultivation in Finland release around 22 ton-CO_2−eq_/ha/year. Depending on drainage intensity and management, forestry-related emissions range from net sequestration of −8 ton-CO_2−eq_/ha/year to emissions of 10 ton-CO_2−eq_/ha/year. Byrne, et al.^65^ report that high-emission regions in European peatlands—the Southeast Mediterranean, Germany, and the Netherlands—exhibit emissions exceeding 12.8 ton-CO_2__-eq_/ha/year, primarily due to intensive agricultural use. Meanwhile, intact peatlands in Finland and the UK emit less than 4.04 ton-CO_2__-eq_/ha/y. Significant differences in estimates occurred between the RS approach and the common approach: the RS-based approach calculates an annual emission of 7.49 tons CO_2-eq_ per hectare, while the common approach estimates 14.49 ton-CO_2__-eq_/ha/year (Table 2; Fig. 6).

**Table 2:** Summary of Estimation of the annual C emission (ton-CO_2−eq_/ha/year) obtained from different approaches.

	The RS approach	Common approach
*C emission rate * *(ton-CO* *2* *-eq/ha/year)*	***7.49***	***14.49***

## Conclusions

This study lays the foundation for developing a comprehensive, fully remote sensing-based approach to improve the accuracy of peatland C emission estimates using peat subsidence as a proxy. By utilizing subsidence rates, peat properties, and the oxidation component, this method makes C emission estimation more accessible to researchers and managers. Although the presented methodology requires calibration elsewhere, the computational tool we presented and calibrated to the actual conditions of an extensive peatland seems ready for application in every possible peatland in the world, opening up the field for large-scale analysis in areas where field studies are significantly hampered (e.g., the vast peatlands of Canada and the West Siberian Plain). In these places, the uncertainty of the developed method gives way to the scale of the carbon emission and accumulation phenomenon being assessed, providing the appropriate order of magnitude of the assessed process. Refining this algorithm requires collaboration among experts with access to in-situ data and specialized knowledge of each parameter. A critical step in this improvement is evaluating the accuracy of acquisition methods for oxidation components, BD, and SOC. The primary objective of this study was, however, not only to propose an approach for estimating C emissions using subsidence as a proxy but also to assess the reliability of key parameters. The results indicate that the estimated C emission rate for the case study was 7.49 ton-CO_2-eq_/ha/year using the proposed framework, compared to 14.49 ton-CO_2-eq_/ha/year obtained from the conventional approach. The normalized differences of 0.133 for SOC and 0.0865 for BD further support the reliability of this method for assessing peat properties. Nevertheless, further research is needed to enhance the accuracy of C emission estimates derived from peatland subsidence.

## Materials and methods

### Study area

**Fig. 7:**
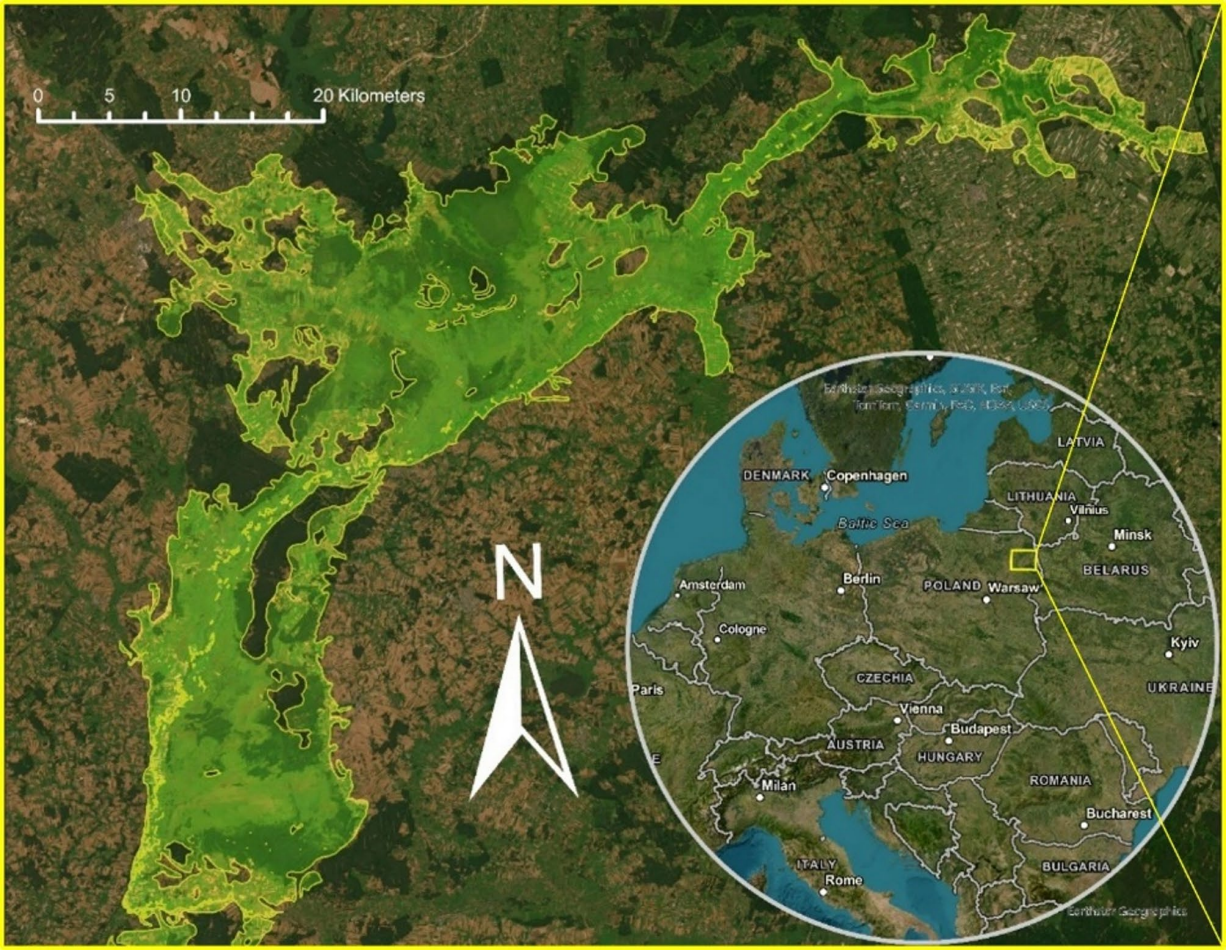
Location of the Biebrza Valley, 53^o^12′49″N-53^o^44′45″N and 22^o^26′00″E-23^o^30′44″E, in northeastern Poland. Source of background map: Esri World Topographic Map (accessed on 20.06.2025)

Biebrza Valley, located in northeastern Poland (Fig. 7), is an ecologically diverse region renowned for its glacial-formed peat landscape, which is covered with a peat layer reaching a max 8 m thickness and rich biodiversity^66,67^. The valley encompasses a variety of ecosystems, including peatlands, floodplains, and marshes, making it one of the most significant peatland complexes in Europe^68^ providing a critical habitat for numerous unique plant and animal species^69^. Recently, Biebrza Valley has faced significant anthropogenic pressures, primarily through land use changes and drainage measures intended to boost agricultural productivity. These interventions have led to subsidence phenomena, where the draining of peatlands has caused the ground to sink, presenting considerable environmental challenges^70^. The climatic conditions in Biebrza Valley are characterized by a mean annual temperature of around 6.6 °C^71^ and a mean annual precipitation of 570 mm (ranging from 470 to 730 mm)^72^. Based on more than a decade of groundwater level measurements, a general decreasing trend of −0.21 m has been observed^73^. In the Biebrza Basin, vary in thickness and stratigraphy depending on the type and extent of water supply. Peats in the 1–2 m thickness range predominate, while the deepest thickness of the peat layer reaches over 6 m.

### The relationship between C emission and peat subsidence

The annual C emissions from peatland oxidation through a peatland subsidence proxy can be quantified using Eq. (1), expressed in units of tons of CO₂-equivalent per hectare per year. Any other C emissions through loss of peat through for example, erosion, are not part of this equation:

1 $$\:C\:emission={\alpha\:\varDelta\:h}_{s}\times\:{\rho\:}_{peat}\times\:{f}_{OC}\times\:3.67\times\:{10}^{-2}$$

where:C emission: The rate of Carbon loss (ton CO_2-eq_/ha/year); α∆h_s_: The rate of Peat loss, oxidation component of subsidence, (m⁄year); *ρ*_peat_: Bulk density (kg⁄m^3^ );  *f*_*OC*_: Soil organic carbon content (g-C/kg); 3.67: The conversion factor from C to CO_2_; 10^−2^: The conversion factor from g/m^2^ to ton/ha

Equation (1) is employed to estimate C emissions resulting from peat subsidence by incorporating the subsidence rate ($\:{\varDelta\:h}_{s}$), the oxidation component ($\:\alpha\:)$, and the peat property (C_v_)—including the BD (*ρ*_peat_) and SOC content (*f*_*OC*_). This study uses CO₂-equivalent (CO_2-eq_) to represent C emission because it provides a straightforward and comparable measure of greenhouse gas emissions. It is necessary to mention that the term CO_2-eq_ is explicitly used to denote the mass conversion of elemental C to carbon dioxide (CO₂) based on molecular weight differences, applying a factor of 3.67. This factor accounts for the ratio of CO₂ (44 g/mol) to C (12 g/mol), representing the complete oxidation of C to CO₂. In climate science, CO_2-eq_ is often used to express the global warming potential (GWP) of various greenhouse gases relative to CO₂. However, in the context of this study, CO_2-eq_ strictly refers to the mass-based conversion and should not be confused with GWP-based equivalence. This approach aligns with the fundamental principles of the POXAPS (Peat OXidation And Permanent Shrinkage) model, which estimates the contribution of oxidation and shrinkage to the overall subsidence^31,43^. The POXAPS model elucidates the relationship between the decomposition of organic matter and CO₂ emissions^50,74^. Previous studies have supported the use of this equation to estimate CO₂ emissions in both northern and tropical peatlands^41–48^.

### Acquisition of Peat Subsidence Rate (*Δh*_*s*_)

This study employs the remote sensing-based approach introduced by Ghezelayagh, et al.^57^ to estimate peat surface vertical displacements. This method utilizes interferometric synthetic aperture radar (InSAR) techniques, leveraging Sentinel-1 satellite data processed through the ASF OnDemand InSAR cloud computing platform. To enhance the accuracy of interferometric measurements, the methodology incorporates a systematic error reduction framework that accounts for key factors, including satellite positioning, atmospheric conditions, vegetation cover, and temporal spacing between radar acquisitions. A detailed explanation of this approach is provided in Ghezelayagh, et al.^57^.

### Acquisition of peat properties (Cv)

There are three approaches to assess peat properties (Cv) (BD and SOC) in this study. These three approaches provide different methods for obtaining peat properties, each with its own set of advantages and limitations. *Field-based sampling* offers high accuracy but is resource-intensive; *the RS framework*—the approach proposed in this study—provides a broad and efficient alternative but may require validation against ground data; *the common approach* offers simplicity and consistency based on established research but may not be accurate enough. In this study, we used the results obtained from the Field-based sampling approach as a criterion for validating two other approaches.

- Constant value (a common approach in peatland studies).

In peatland studies, a common approach is to use a constant value for BD and SOC content, typically held constant at 0.08 g/cm³ (80 kg/m³) and 55% (550 g-C/kg), respectively^31,75,76^.

- RS framework approach.

In northern boreal and temperate peatlands, an alternative approach for acquiring bulk density (BD) and soil organic carbon (SOC) data can be achieved by utilizing the comprehensive dataset compiled by Loisel, et al.^56^. This dataset provides BD and SOC values derived from an extensive collection of peat core samples, offering a robust and widely applicable reference for similar environmental studies. The dataset includes a synthesis of peatland soil properties constructed across 215 sites located north of 45°N. The values provided encompass different peatland types, including ombrotrophic bogs, minerotrophic fens, and permafrost-affected peatlands, making it a valuable resource for estimating soil properties in various case studies. BD and SOC values can be obtained by identifying the closest comparable peatland sites within the dataset (Table 3) in terms of geographical location, climatic conditions, and peatland type. As shown in Table 3, BD and SOC values can be extracted for the most relevant peat cores that match the locational, ecological, and hydrological conditions of the study area. These values were then applied to the case study region to estimate BD and SOC without needing direct field sampling. These values are also acquired from the study by Agus, et al.^49^ for tropical peatlands in which they used 1826 in-situ field sampling.

Indeed, instead of conducting in-situ field sampling and laboratory analysis to determine BD and SOC, these values are extracted from the dataset developed by Loisel, et al.^56^ for northern boreal and temperate peatlands and Agus, et al.^51^ for tropical peatlands. Utilizing an existing dataset eliminates the need for extensive fieldwork and laboratory processing, making it an effective approach for studies constrained by logistical or financial limitations. By demonstrating the applicability of this dataset for BD and SOC estimation, it provides a replicable framework for its use in other regions where direct field data may be unavailable. It is necessary to mention that these values should be used in the framework only if on-site in situ information is missing or largely incomplete. In cases where accurate in situ data exist, using those data is the preferred option.

Future research can expand on this methodology by integrating additional datasets, remote sensing techniques, or machine learning models to refine BD and SOC estimations across diverse peatland environments. Incorporating remote sensing data could further enhance the spatial precision of BD and SOC predictions, allowing for more detailed peatland carbon stock assessments.

**Table 3:** Peat properties by regions. Means, standard deviations, and the number of samples in parentheses (n) are presented. Northern peatland data derived from loisel, et al.^56^ whereas tropical peatland data sourced from and agus, et al.^51^.

Regions	Bulk density (kg/m^3^)	Soil Organic Carbon content (g-C/kg)	
Alaska	168 ± 87 (*n* = 1659)		424 ± 37 (64)
Western Canada	166 ± 76 (3635)		450 ± 43 (382)
*Hudson Bay and James Bay*	*97 ± 38 (6002)*		*479 ± 45 (1026)*
*Eastern Canada/United States*	*100 ± 39 (2834)*		*489 ± 37 (1084)*
*Western European Islands*	*55 ± 27 (656)*		*540 ± 25 (242)*
*Continental Europe*	*120 ± 139 (410)*		*389 ± 13 (60)*
*Fennoscandia*	*75 ± 43 (562)*		*444 ± 57 (580)*
*Western Russia*	*118 ± 70 (2701)*		*492 ± 32 (74)*
*Eastern Russia and Asia*	*116 ± 63 (2761)*		*360 ± 92 (229)*
*Tropical peatlands*	*126.67 (1826)*		*515.8 (1826)*

### Acquisition of Oxidation component (α)

By modifying the equations from Ma, et al.^55^ we derived the equations in Table 1. Ma and colleagues’ study established links between peat subsidence rate, drainage duration (years since drainage), and the oxidation component (the proportion of subsidence attributed to peat oxidation) across various climate zones and land use.

For instance, in agricultural areas, they found that the peat subsidence rate (Δh_s_​) can be determined using Eq. 2 while the oxidation component (α) is given by Eq. 3.

2 $$\:{\varDelta\:h}_{s}={\left(13.95\pm\:0.98\right){T}_{d}}^{(-0.58\pm\:0.03)}$$

where:

$$\:{\varDelta\:h}_{s}:\:\:\:\:Peat\:Subsidence\:Rate\:\left(cm/year\right)$$ $$\:{T}_{d}:\:\:\:\:\:\:Drainage\:duration\:\left(1/year\right)$$

3 $$\alpha\:\:=\left(2.15\pm\:2.79\right)+\left(12.05\pm\:0.79\right){ln}({T}_{d})$$

where:

$$\:\alpha\::\:\:\:\:\:\:\:\:Oxidation\:compponent\:\left(\%\right)$$ $$\:{T}_{d}:\:\:\:\:\:\:Drainage\:duration\:\left(1/year\right)$$

From these equations, we derived additional formulations. Equation 4 is obtained by rearranging Eq. 2, while Eq. 5 results from substituting Eq. 4 into Eq. 3. This allows the oxidation component to be directly estimated using the peat subsidence rate:

4 $$\:{T}_{d}=\frac{\sqrt[-0.58]{{\varDelta\:h}_{s}}}{13.95}\:$$

5 $$\:\alpha\:=\left(2.15\pm\:2.79\right)+\left(12.05\pm\:0.79\right){ln}(\frac{\sqrt[-0.58]{{\varDelta\:h}_{s}}}{13.95})$$

Following a similar procedure, the equations presented in Table 1 were derived from those in Table 4.

**Table 4:** Equations illustrating relationships between the peat subsidence rate ($\:{\varDelta\:h}_{s},\:cm/year$), drainage duration ($\:{T}_{d},\:1/year$), and oxidation component (α, %) across different peatland types, as presented by ma, et al.^55^.

Peatland type		Equations	
*Northern boreal*	***Agriculture***	$\:{\varDelta\:h}_{s}={\left(13.95\pm\:0.98\right){T}_{d}}^{(-0.58\pm\:0.03)}$	*α* $\:\:=\left(2.15\pm\:2.79\right)+\left(12.05\pm\:0.79\right){ln}({T}_{d})$
	***Forestry***	$\:{\varDelta\:h}_{s}={\left(5.36\pm\:0.35\right){T}_{d}}^{(-0.83\pm\:0.09)}$	
	***Grassland***	$\:{\varDelta\:h}_{s}={\left(5.55\pm\:0.43\right){T}_{d}}^{(-0.36\pm\:0.04)}$	
*Tropical*	***Agriculture***	$\:{\varDelta\:h}_{s}={\left(6.63\pm\:0.25\right){T}_{d}}^{(-0.37\pm\:0.02)}$	*α* $\:\:=\left(37.05\pm\:3.84\right)+\left(14.36\pm\:1.63\right){ln}({T}_{d})$
	***Forestry***		

## Supplementary Information

Below is the link to the electronic supplementary material.

Supplementary Material 1.

## Data Availability

The single-look complex (SLC) Sentinel-1 imagery and associated metadata are available at the Alaska Satellite Facility’s Vertex Portal. The original data can be obtained from the corresponding author upon reasonable request.

## References

[1] Abrahms, B. et al. Climate change as a global amplifier of human–wildlife conflict. *Nat. Clim. Change*. **13**, 224–234. 10.1038/s41558-023-01608-5 (2023).

[2] Berrang-Ford, L. et al. A systematic global stocktake of evidence on human adaptation to climate change. *Nat. Clim. Change*. **11**, 989–1000. 10.1038/s41558-021-01170-y (2021).

[3] Darwin, R. Effects of greenhouse gas emissions on world agriculture, food consumption, and economic welfare. *Clim. Change*. **66**, 191–238. 10.1023/B:CLIM.0000043138.67784.27 (2004).

[4] Lu, L. C., Chiu, S. Y., Chiu, Y. & Chang, T. H. Sustainability efficiency of climate change and global disasters based on greenhouse gas emissions from the parallel production sectors – A modified dynamic parallel three-stage network DEA model. *J. Environ. Manage.* **317**, 115401. 10.1016/j.jenvman.2022.115401 (2022). 35660833 10.1016/j.jenvman.2022.115401

[5] Bajoria, A., Kanpariya, J. & Bera, A. in *Advances and Technology Development in Greenhouse Gases: Emission, Capture and Conversion* (eds Mohammad Reza Rahimpour, Mohammad Amin Makarem, & Maryam Meshksar) 121–135 (Elsevier, 2024).

[6] Montzka, S. A., Dlugokencky, E. J. & Butler, J. H. Non-CO2 greenhouse gases and climate change. *Nature* **476**, 43–50. 10.1038/nature10322 (2011). 21814274 10.1038/nature10322

[7] IPCC. in. *Climate Change 2021 – The Physical Science Basis: Working Group I Contribution To the Sixth Assessment Report of the Intergovernmental Panel on Climate Change (ed Change Intergovernmental Panel on Climate) 2215–2256* (Cambridge University Press, 2023).

[8] Ayres, R. U. & Walter, J. The greenhouse effect: damages, costs and abatement. *Environ. Resource Econ.* **1**, 237–270. 10.1007/BF00367920 (1991).

[9] Jones, M. W. et al. National contributions to climate change due to historical emissions of carbon dioxide, methane, and nitrous oxide since 1850. *Sci. Data*. **10**, 155. 10.1038/s41597-023-02041-1 (2023). 36991071 10.1038/s41597-023-02041-1 PMC10060593

[10] Rogelj, J., Forster, P. M., Kriegler, E., Smith, C. J. & Séférian, R. Estimating and tracking the remaining carbon budget for stringent climate targets. *Nature* **571**, 335–342. 10.1038/s41586-019-1368-z (2019). 31316194 10.1038/s41586-019-1368-z

[11] Rogelj, J. & Lamboll, R. D. Substantial reductions in non-CO2 greenhouse gas emissions reductions implied by IPCC estimates of the remaining carbon budget. *Commun. Earth Environ.* **5**, 35. 10.1038/s43247-023-01168-8 (2024).

[12] Tian, H. et al. The terrestrial biosphere as a net source of greenhouse gases to the atmosphere. *Nature* **531**, 225–228. 10.1038/nature16946 (2016). 26961656 10.1038/nature16946

[13] Schulze, E. D. et al. Importance of methane and nitrous oxide for europe’s terrestrial greenhouse-gas balance. *Nat. Geosci.* **2**, 842–850. 10.1038/ngeo686 (2009).

[14] Tian, H. et al. North American terrestrial CO2 uptake largely offset by CH4 and N2O emissions: toward a full accounting of the greenhouse gas budget. *Clim. Change*. **129**, 413–426. 10.1007/s10584-014-1072-9 (2015). 26005232 10.1007/s10584-014-1072-9 PMC4439729

[15] Vitt, D. H. & Short, P. in *Wetlands and Habitats* 27–36CRC Press, (2020).

[16] Archibold, O. & Archibold, O. Terrestrial wetlands. *Ecology World Vegetation*, 319–353 (1995).

[17] Minasny, B. et al. Digital mapping of peatlands – A critical review. *Earth Sci. Rev.* **196**, 102870. 10.1016/j.earscirev.2019.05.014 (2019).

[18] Liu, W., Fritz, C., van Belle, J. & Nonhebel, S. Production in peatlands: comparing ecosystem services of different land use options following conventional farming. *Sci. Total Environ.* **875**, 162534. 10.1016/j.scitotenv.2023.162534 (2023). 36878291 10.1016/j.scitotenv.2023.162534

[19] Omar, M. S. et al. Peatlands in Southeast asia: A comprehensive geological review. *Earth Sci. Rev.* **232**, 104149. 10.1016/j.earscirev.2022.104149 (2022).

[20] Humpenöder, F. et al. Peatland protection and restoration are key for climate change mitigation. *Environ. Res. Lett.* **15**, 104093. 10.1088/1748-9326/abae2a (2020).

[21] Page, S. E. & Baird, A. J. Peatlands and global change: response and resilience. *Annu. Rev. Environ. Resour.* **41**, 35–57. 10.1146/annurev-environ-110615-085520 (2016).

[22] Beaulne, J., Garneau, M., Magnan, G. & Boucher, É. Peat deposits store more carbon than trees in forested peatlands of the boreal biome. *Sci. Rep.* **11**, 2657. 10.1038/s41598-021-82004-x (2021). 33514778 10.1038/s41598-021-82004-x PMC7846601

[23] Loisel, J. et al. Insights and issues with estimating Northern peatland carbon stocks and fluxes since the last glacial maximum. *Earth Sci. Rev.* **165**, 59–80. 10.1016/j.earscirev.2016.12.001 (2017).

[24] Loisel, J. et al. Expert assessment of future vulnerability of the global peatland carbon sink. *Nat. Clim. Change*. **11**, 70–77. 10.1038/s41558-020-00944-0 (2021).

[25] Strack, M., Davidson, S. J., Hirano, T. & Dunn, C. The potential of peatlands as Nature-Based climate solutions. *Curr. Clim. Change Rep.* **8**, 71–82. 10.1007/s40641-022-00183-9 (2022).

[26] Kalhori, A. et al. Temporally dynamic carbon dioxide and methane emission factors for rewetted peatlands. *Commun. Earth Environ.* **5**, 62. 10.1038/s43247-024-01226-9 (2024).

[27] Fortuniak, K. et al. Methane and carbon dioxide fluxes of a temperate mire in central Europe. *Agric. For. Meteorol.* **232**, 306–318. 10.1016/j.agrformet.2016.08.023 (2017).

[28] Harris, L. I. et al. The essential carbon service provided by Northern peatlands. *Front. Ecol. Environ.* **20**, 222–230. 10.1002/fee.2437 (2022).

[29] Leifeld, J. & Menichetti, L. The underappreciated potential of peatlands in global climate change mitigation strategies. *Nat. Commun.* **9**, 1071. 10.1038/s41467-018-03406-6 (2018). 29540695 10.1038/s41467-018-03406-6 PMC5851997

[30] He, H. & Roulet, N. T. Improved estimates of carbon dioxide emissions from drained peatlands support a reduction in emission factor. *Commun. Earth Environ.* **4**, 436. 10.1038/s43247-023-01091-y (2023).

[31] Hoyt, A. M., Chaussard, E., Seppalainen, S. S. & Harvey, C. F. Widespread subsidence and carbon emissions across Southeast Asian peatlands. *Nat. Geosci.* **13**, 435–440. 10.1038/s41561-020-0575-4 (2020).

[32] Marshall, C., Bradley, A. V., Andersen, R. & Large, D. J. Using peatland surface motion (bog breathing) to monitor peatland action sites. *NatureScot Res. Rep.* **1269**. (2021).

[33] Morton, P. A. & Heinemeyer, A. Bog breathing: the extent of peat shrinkage and expansion on blanket bogs in relation to water table, Heather management and dominant vegetation and its implications for carbon stock assessments. *Wetlands Ecol. Manage.* **27**, 467–482. 10.1007/s11273-019-09672-5 (2019).

[34] van Asselen, S. et al. The relative contribution of peat compaction and oxidation to subsidence in built-up areas in the Rhine-Meuse delta, the Netherlands. *Sci. Total Environ.* **636**, 177–191. 10.1016/j.scitotenv.2018.04.141 (2018). 29704713 10.1016/j.scitotenv.2018.04.141

[35] Huning, L. S. et al. Global land subsidence: impact of climate extremes and human activities. *Rev. Geophys.* **62**10.1029/2023RG000817 (2024). e2023RG000817.

[36] Oliveira, B. R. F., Smit, M. P. J., van Paassen, L. A., Grotenhuis, T. C. & Rijnaarts, H. H. M. Functional properties of soils formed from biochemical ripening of dredged sediments—subsidence mitigation in delta areas. *J. Soils Sediments*. **17**, 286–298. 10.1007/s11368-016-1570-7 (2017).

[37] Samuel, M. K. & Evers, S. L. The role of compaction on pyhsicochemical properties and carbon emissions of tropical peat soils: a review. *Jurnal Teknologi*. **85**, 83–96. 10.11113/jurnalteknologi.v85.18340 (2023).

[38] Shiraishi, T., Hirata, R., Hayashi, M. & Hirano, T. Carbon dioxide emissions through land use change, fire, and oxidative peat decomposition in Borneo. *Sci. Rep.* **13**, 13067. 10.1038/s41598-023-40333-z (2023). 37567930 10.1038/s41598-023-40333-z PMC10421864

[39] Seidel, R., Dettmann, U. & Tiemeyer, B. Reviewing and analyzing shrinkage of peat and other organic soils in relation to selected soil properties. *Vadose Zone J.* **22**, e20264. 10.1002/vzj2.20264 (2023).

[40] Zanello, F., Teatini, P., Putti, M. & Gambolati, G. Long term peatland subsidence: experimental study and modeling scenarios in the Venice Coastland. *J. Geophys. Research: Earth Surf.* **116**10.1029/2011JF002010 (2011).

[41] Sari, N., Hayati, N., Uzzulfa, M. A., Arief, R. & Krisna, T. C. Mapping carbon dioxide (CO < sub > 2) emissions from peat subsidence using carbon parameters and InSAR observations in South kalimantan, Indonesia. *Soil. Sci. Annual*. **74**, 1–10. 10.37501/soilsa/169656 (2023).

[42] Khodaei, B., Hashemi, H., Salimi, S. & Berndtsson, R. Substantial carbon sequestration by peatlands in temperate areas revealed by InSAR. *Environ. Res. Lett.* **18**, 044012. 10.1088/1748-9326/acc194 (2023).

[43] Hayati, N. et al. *Int. Arch. Photogramm. Remote Sens. Spatial Inf. Sci.* XLIII-B3- 277–284, (2022). 10.5194/isprs-archives-XLIII-B3-2022-277-2022 (2022).

[44] Erkens, G., Van der Meulen, M. J. & Middelkoop, H. Double trouble: subsidence and CO2 respiration due to 1,000 years of Dutch coastal peatlands cultivation. *Hydrogeol. J.* **24**, 551 (2016).

[45] Zhou, Z., Li, Z., Waldron, S. & Tanaka, A. in *IEEE International Geoscience and Remote Sensing Symposium (IGARSS).* 6797–6798. 6797–6798. (2016).

[46] Hooijer, A. et al. Subsidence and carbon loss in drained tropical peatlands. *Biogeosciences* **9**, 1053–1071. 10.5194/bg-9-1053-2012 (2012).

[47] Mos, H. et al. Differences in CO2 emissions on a Bare-Drained peat area in sarawak, malaysia, based on different measurement techniques. *Agriculture* **13**, 622 (2023).

[48] Anshari, G. Z., Gusmayanti, E. & Novita, N. The use of subsidence to estimate carbon loss from deforested and drained tropical peatlands in Indonesia. *Forests* **12**, 732 (2021).

[49] Couwenberg, J., Dommain, R. & Joosten, H. Greenhouse gas fluxes from tropical peatlands in south-east Asia. *Glob. Change Biol.* **16**, 1715–1732. 10.1111/j.1365-2486.2009.02016.x (2010).

[50] Wösten, J. H. M., Ismail, A. B. & van Wijk, A. L. M. Peat subsidence and its practical implications: a case study in Malaysia. *Geoderma* **78**, 25–36. 10.1016/S0016-7061(97)00013-X (1997).

[51] Agus, F., Hairiah, K. & Mulyani, A. *Measuring carbon stock in peat soils: practical guidelines*. (Citeseer, (2010).

[52] Page, S. E. et al. Review of peat surface greenhouse gas emissions from oil palm plantations in Southeast Asia. *ICCT White Paper*. **15**, 1–78 (2011).

[53] Grønlund, A., Hauge, A., Hovde, A. & Rasse, D. P. Carbon loss estimates from cultivated peat soils in norway: a comparison of three methods. *Nutr. Cycl. Agrosyst.* **81**, 157–167. 10.1007/s10705-008-9171-5 (2008).

[54] Schothorst, C. J. Subsidence of low Moor peat soils in the Western Netherlands. *Geoderma* **17**, 265–291. 10.1016/0016-7061(77)90089-1 (1977).

[55] Ma, L. et al. A globally robust relationship between water table decline, subsidence rate, and carbon release from peatlands. *Commun. Earth Environ.* **3**, 254. 10.1038/s43247-022-00590-8 (2022).

[56] Loisel, J. et al. A database and synthesis of Northern peatland soil properties and holocene carbon and nitrogen accumulation. *Holocene* **24**, 1028–1042. 10.1177/0959683614538073 (2014).

[57] Ghezelayagh, P. et al. Developing a remote-sensing-based indicator for peat soil vertical displacement. A case study in the Biebrza valley, Poland. *Ecol. Ind.* **166**, 112305. 10.1016/j.ecolind.2024.112305 (2024).

[58] Hrysiewicz, A. et al. Estimation and validation of InSAR-derived surface displacements at temperate Raised peatlands. *Remote Sens. Environ.* **311**, 114232. 10.1016/j.rse.2024.114232 (2024).

[59] Ghezelayagh, P., Eini, M. R. & Grygoruk, M. Assessing carbon accumulation through peat vertical displacement: the influence of climate and land use across diverse peatland characteristics. *Sci. Total Environ.* **958**, 178132. 10.1016/j.scitotenv.2024.178132 (2025). 39693648 10.1016/j.scitotenv.2024.178132

[60] Gnatowski, T., Szatyłowicz, J., Brandyk, T. & Kechavarzi, C. Hydraulic properties of Fen peat soils in Poland. *Geoderma* **154**, 188–195. 10.1016/j.geoderma.2009.02.021 (2010).

[61] Leifeld, J., Müller, M. & Fuhrer, J. Peatland subsidence and carbon loss from drained temperate Fens. *Soil Use Manag.* **27**, 170–176. 10.1111/j.1475-2743.2011.00327.x (2011).

[62] Wilson, D. et al. Derivation of greenhouse gas emission factors for peatlands managed for extraction in the Republic of Ireland and the united Kingdom. *Biogeosciences* **12**, 5291–5308. 10.5194/bg-12-5291-2015 (2015).

[63] Hiraishi, T. et al. 2013 supplement to the 2006 IPCC guidelines for national greenhouse gas inventories: Wetlands. *IPCC, Switzerland* (2014).

[64] Höper, H. et al. *In Peatlands and Climate Change*182–210 (International Peat Society, 2008).

[65] Byrne, K. A. et al. EU peatlands: Current carbon stocks and trace gas fluxes. (2004).

[66] Grygoruk, M. & Rannow, S. Mind the gap! Lessons from science-based stakeholder dialogue in climate-adapted management of wetlands. *J. Environ. Manage.* **186**, 108–119. 10.1016/j.jenvman.2016.10.066 (2017). 27823904 10.1016/j.jenvman.2016.10.066

[67] Grygoruk, M., Kochanek, K. & Mirosław-Świątek, D. Analysis of long-term changes in inundation characteristics of near-natural temperate riparian habitats in the lower basin of the Biebrza valley, Poland. *J. Hydrology: Reg. Stud.* **36**, 100844. 10.1016/j.ejrh.2021.100844 (2021).

[68] Stachowicz, M., Venegas-Cordero, N. & Ghezelayagh, P. Two centuries of changes - revision of the hydrography of the Biebrza valley, its transformation and probable ecohydrological challenges. *Ecohydrol. Hydrobiol.* **24**, 738–748. 10.1016/j.ecohyd.2023.08.008 (2024).

[69] Budka, M., Jobda, M., Szałański, P. & Piórkowski, H. Effect of agri-environment measure for the aquatic warbler on bird biodiversity in the extensively managed landscape of Biebrza marshes (Poland). *Biol. Conserv.* **239**, 108279. 10.1016/j.biocon.2019.108279 (2019).

[70] Oleszczuk, R., Zając, E. & Urbański, J. Verification of empirical equations describing subsidence rate of peatland in central Poland. *Wetlands Ecol. Manage.* **28**, 495–507. 10.1007/s11273-020-09727-y (2020).

[71] Sulwiński, M., Mętrak, M., Wilk, M. & Suska-Malawska, M. Smouldering fire in a nutrient-limited wetland ecosystem: Long-lasting changes in water and soil chemistry facilitate shrub expansion into a drained burned Fen. *Sci. Total Environ.* **746**, 141142. 10.1016/j.scitotenv.2020.141142 (2020). 32739756 10.1016/j.scitotenv.2020.141142

[72] Marcinkowski, P., Piniewski, M., Grygoruk, M. & Mirosław-Świątek, D. Climate change in the Biebrza Basin—Projections and ecohydrological implications. *Ecohydrol. Hydrobiol.* **24**, 796–807. 10.1016/j.ecohyd.2024.04.006 (2024).

[73] Stachowicz, M. et al. Estimating mean groundwater levels in peatlands using a bayesian belief network approach with remote sensing data. *Sci. Rev. Eng. Environ. Sci. (SREES)*. **33**, 329–351. 10.22630/srees.9939 (2024).

[74] Dahdal, B. *The Use of Interferometric Spaceborne Radar and GIS To Measure Ground Subsidence in Peat Soils in Indonesia* (University of Leicester, 2011).

[75] Couwenberg, J. & Hooijer, A. Towards robust subsidence-based soil carbon emission factors for peat soils in south-east asia, with special reference to oil palm plantations. *Mires Peat*. **12**, 01 (2013).

[76] Lennartz, B. & Liu, H. Hydraulic functions of peat soils and ecosystem service. *Front. Environ. Sci.* **7**, 92 (2019).

