# Supplementary material for: Towards a remote sensing-based assessment of carbon emissions from peatlands
Source: Sci Rep. 2025 Oct 1;15:34175. doi: 10.1038/s41598-025-15293-1 (PMC12489126; doi:10.1038/s41598-025-15293-1)
Supplement: Supplementary file 1 — Supplementary Material 1. [file 41598_2025_15293_MOESM1_ESM.docx]

# Supplementary material 1:

**Details on InSAR Analysis**

The InSAR technique detects changes in ground surface elevation over specified time periods by creating interferograms, which measure phase differences between reference and secondary radar images. The quality of an interferogram depends on several factors:

1. **Spatial Baseline:** This relates to the satellite’s positions when acquiring the reference and secondary images. Minimizing the perpendicular baseline—i.e., the vertical distance between satellite positions during acquisitions—is crucial for improving interferogram quality.
2. **Atmospheric Moisture:** Variability in atmospheric conditions such as clouds and fog induces phase shifts, potentially causing errors in radar signal measurements.
3. **Surface Cover:** The type and density of ground cover (e.g., vegetation, water bodies) significantly affect the quality of the interferogram.
4. **Temporal Baseline:** Large temporal gaps between radar acquisitions degrade signal quality due to surface noise. Conversely, stacking interferograms from very short temporal baselines may fail to capture slow deformation processes like subsidence. Therefore, longer temporal baselines are essential to detect subtle, gradual motions.

To mitigate these challenges, seasonality and weather conditions are carefully considered when selecting image pairs for interferometric analysis. For this case study, annual interferograms were generated from images acquired during the same month (April) across multiple years, minimizing variability due to vegetation growth and groundwater fluctuations.

Table 1 summarizes the seven selected pairs of annual interferograms used for vertical displacement estimation. All images were acquired in descending orbit mode, using a C-band radar wavelength (5.405 GHz), with an effective spatial resolution of approximately 80 meters (10x2 looks). Data were obtained from the Alaska Satellite Facility (ASF) DAAC on-demand service. Image pairs were optimized by minimizing the perpendicular baseline to enhance data quality.

**Table S1.** Selected pairs of annual interferograms acquired in April. Data obtained from ASF DAAC (2023), comprising modified Copernicus Sentinel data (2021) processed by ESA.

| Pairs | Interval (year) | Date of the reference image | Date of the second-order image | Time interval (days) | P baseline (m) |
| --- | --- | --- | --- | --- | --- |
| 1 | 2015-2016 | 02.04.2015 | 08.04.2016 | 372 | 36.0 |
| 2 | 2016-2017 | 08.04.2016 | 09.04.2017 | 366 | -21.2 |
| 3 | 2017-2018 | 09.04.2016 | 28.04.2018 | 378 | 14.5 |
| 4 | 2018-2019 | 04.04.2018 | 29.04.2019 | 390 | 8.7 |
| 5 | 2019-2020 | 11.04.2019 | 11.04.2020 | 366 | 6.5 |
| 6 | 2020-2021 | 11.04.2020 | 06.04.2021 | 360 | -18.9 |
| 7 | 2021-2022 | 06.04.2021 | 13.04.2022 | 372 | 38.8 |
